# Insulin Modulates *Paracoccidioides brasiliensis*-Induced Inflammation by Restoring the Populations of NK Cells, Dendritic Cells, and B Lymphocytes in Lungs

**DOI:** 10.1155/2018/6209694

**Published:** 2018-10-22

**Authors:** Felipe Beccaria Casagrande, Sabrina de Souza Ferreira, Fernanda Peixoto Barbosa Nunes, Lavínia Maria Dal'Mas Romera, Suelen Silvana dos Santos, Fernando Henrique Galvão Tessaro, Paula Regina Knox de Souza, Sandro Rogério Almeida, Joilson Oliveira Martins

**Affiliations:** ^1^Laboratory of Immunoendocrinology, Department of Clinical and Toxicological Analyses, School of Pharmaceutical Sciences of University Sao Paulo (FCF/USP), São Paulo, Brazil; ^2^Laboratory of Mycology, Department of Clinical and Toxicological Analyses, FCF/USP, São Paulo, Brazil; ^3^Universidade Paulista (UNIP), São Paulo, Brazil

## Abstract

Paracoccidioidomycosis, a key issue for Brazilian health service, can be aggravated in patients with impaired immunological responses, such as diabetic patients. We evaluated the role of insulin in inflammatory parameters in diabetic and nondiabetic mice using a systemic mycosis *Paracoccidioides brasiliensis* (Pb) model. Diabetic C57BL-6 mice and controls were infected with Pb18 and treated with insulin for 12 days prior to experiments. After 55 days, infected diabetic mice exhibited fewer leukocytes in both peritoneal lavage fluid (PeLF) and bronchoalveolar lavage fluid and reduced secretion of interleukin- (IL-) 6 in lungs. In addition, diabetic mice presented a reduced influx of TCD4+ cells, TCD8+ cells, B lymphocytes, NK cells, and dendritic cells compared to control infected groups. Insulin treatment restored the leukocyte number in PeLF and restored the presence of B lymphocytes, dendritic cells, and NK cells in lungs of diabetic animals. The data suggest that diabetic mice present impaired immunological response to Pb18 infection and insulin modulates inflammation by reducing IL-6 levels in lung and CINC-1 levels in spleen and liver homogenates, restoring leukocyte concentrations in PeLF and also restoring populations of dendritic cells and B lymphocytes in lungs of diabetic mice, permitting the host to better control the infection.

## 1. Introduction

Systemic mycoses are infections caused by pathogenic fungi or fungi whose pathogenicity increases due to immune system impairment. In Brazil, systemic mycoses are among the ten main causes of death due to infectious diseases [1]. Paracoccidioidomycosis (PCM) is a systemic mycosis caused by the dimorphic fungi *Paracoccidioides brasiliensis* (Pb) and *Paracoccidioides lutzii* [2–5]. PCM is endemic in Latin America, and it has a higher prevalence in South America, where it primarily affects rural workers. Once inhaled by the host, the fungus interacts with alveolar macrophages and dendritic cells present in the lungs, where it can form a pulmonary focus or spread through the blood and lymphatic vessels [6]. Survival of the fungus inside the host's macrophages is essential for successful infection and depends on several features: the strain of the fungus, its responsiveness to external factors such as temperature changes and oxidative stress, and the efficiency of the host's immunological response [7]. As observed in other systemic mycoses, the most relevant response against PCM is cellular immunity mediated by interferon- (IFN-) *γ* during macrophage activation [8–10]. The activation of macrophages leads to the production of T helper 1 (Th1) inflammatory cytokines such as tumor necrosis factor- (TNF-) *α*, interleukin- (IL-) 1*β*, IL-6, and IL-12, whereas limited activation develops into a Th2 response (IL-4 secretion) [11–13]. It is also known that an effective response against Pb depends on both TCD4 and TCD8 lymphocyte activation in the presence of ideal concentrations of Th1 inflammatory cytokines [12–14]. Murine models of PCM have been used to study the several aspects of Pb infection, the outcomes varying from cure to a progressive granulomatous disease, according to the susceptibility of the strain of mice [15].

*Diabetes mellitus* (DM) represents a group of metabolic diseases related to defective production, secretion, or activity of insulin resulting in hyperglycemia associated with deleterious effects on the life quality of patients [16, 17], and it is frequently associated with complications that lead to multiple organ dysfunction. Previous studies have shown a relationship between DM and a high prevalence of infections in mucosa and airways [18, 19]. Reduced levels of TNF-*α* and IL-1*β* and decreased expression of intercellular adhesion molecule- (ICAM-) 1, resulting in diminished interactions between the leukocyte and endothelium and reduced migration of phagocytes to inflammatory sites, have been suggested as possible causes for this increased susceptibility [19–21]. It was also shown that phagocytosis of zymosan particles and hydrogen peroxide production, which are both reduced in peritoneal macrophages of diabetic rats, were restored by a single dose of insulin [22], suggesting an important role of the hormone in the phagocytic activity of neutrophils and macrophages.

Systemic mycoses such as PCM are key issues of the regarding Brazilian health service, and it can be aggravated in patients with impaired immunological responses such as diabetic patients. On the other hand, the number of diabetic patients is growing, and projections for 2040 have estimated a 55% increase over the already significant population [23]. Expanding the existent knowledge of inflammatory and immunological responses initiated by Pb infection in diabetic patients is highly relevant. Thus, this work is aimed at evaluating the influence of insulin treatment on systemic mycosis caused by *Paracoccidioides brasiliensis* in a murine model of insulin deficiency (type 1 *diabetes mellitus*, DM1) induced by alloxan.

## 2. Material and Methods

### 2.1. Animals

We used specific pathogen-free male 8- to 12-week-old C57BL/6 mice weighing 18–22 g at the beginning of the experimental period. This strain of mice was chosen for presenting an intermediary susceptibility to PCM [15]. The animals were maintained in a controlled environment at 22°C and under a 12 h light-dark cycle and were allowed *ad libitum* access to food and water throughout the observation period. This study was carried out in strict accordance with the principles and guidelines adopted by the Brazilian National Council for the Control of Animal Experimentation (CONCEA) and approved by the Ethical Committee on Animal Use (CEUA) of the School of Pharmaceutical Sciences (FCF) of the University São Paulo (USP) (permit number: CEUA/FCF/421). All surgical procedures were performed under ketamine/xylazine hydrochloride anesthesia, and care was taken to minimize animal suffering.

### 2.2. Induction of Diabetes Mellitus

**Figure 1:**
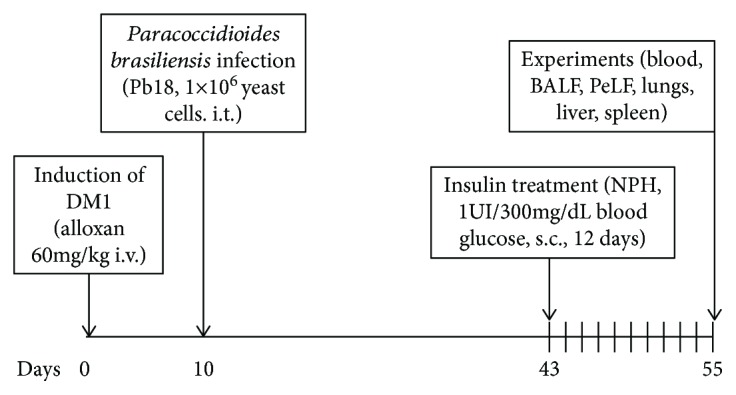
*Paracoccidioides brasiliensis* infection in the *diabetes mellitus* experimental protocol.

Animals were divided into two groups. *Diabetes mellitus* was induced by intravenous injection of alloxan monohydrate (60 mg/kg; Sigma Chemical Co., St. Louis, MO, USA) dissolved in saline solution (0.9% NaCl) [24], whereas mice from the control group were injected with vehicle only. After ten days, the presence of diabetes was verified by blood glucose concentrations higher than 300 mg/dl in blood samples obtained from mouse tails determined using a blood glucose monitor (Accu-Chek Advantage II, Roche Diagnostica, Sao Paulo, SP, Brazil). Variation of body weight was determined by a scale (Adventurer Pro, OHAUS, Toledo, Brazil), where animals were weighted before alloxan administration, ten days after it, and 55 days after it (Figure 1).

### 2.3. *P. brasiliensis* Infection

We used *Paracoccidioides brasiliensis* 18 (Pb18) isolate for this study, which is a strain known to be virulent [25], provided by the Laboratory of Mycology from the Department of Clinical Analysis and Toxicology—FCF-USP. Pb18 yeast cells were maintained in Sabouraud's semisolid culture medium at 37°C and were subcultured weekly. Yeasts from strain Pb18 were collected in a falcon with 10 ml PBS1x (18 mM Na_2_HPO_4_, 3 mM NaH_2_PO_4_·H_2_O, and 140 mM NaCl in Milli-Q water) and homogenized with a bulb pipette. The supernatant was transferred to another falcon with an insulin syringe (BD Biosciences) and posteriorly filtered with a 40 *μ*m cell strainer (BD Biosciences). This step was performed 3 times. The number of cells was counted in the Neubauer chamber.

Mice were anesthetized with ketamine/xylazine hydrochloride and submitted to surgical intratracheal infection with 1 × 10^6^ Pb18 yeast cells in 50 *μ*l sterile PBS. Animals from the control groups received 50 *μ*l sterile PBS only via the same procedure.

### 2.4. Insulin Treatment

Thirty-three days after infection by Pb18 (43 days after induction of DM), diabetic and nondiabetic animals received insulin treatment with neutral protamine Hagedorn insulin (NPH; Eli Lilly, Sao Paulo, SP, Brazil). The glucose levels of the mice from the two treatment groups were measured, and the mice received subcutaneous injections of 2 IU of insulin/600 mg/dl [24] of blood glucose every day at 18:00 h for 12 days [19] (Figure 1).

### 2.5. Hematological Parameters

Blood samples were collected by an intracardiac route. The total number of cells was determined using an automated hematology counter (ABC Vet—Horiba ABX), whereas blood smears were stained with Rosenfeld solution to determine the differential count. A total of 100 cells were counted using a conventional optical microscope (Leica Microsystems, Wetzlar, Germany).

### 2.6. Peritoneal Lavage Fluid (PeLF) and Bronchoalveolar Lavage Fluid (BALF)

After euthanasia, the peritoneum of each mouse was lavaged by repeated injections of 3 ml PBS. The recovered fluid was spun down at 1500 rpm for 10 minutes (Eppendorf). The supernatant was frozen at −70°C for subsequent cytokine measurements. Pelleted cells were resuspended in PBS, and the total number of cells was determined on Neubauer slides. The differential PeLF and BALF counts were obtained after the cells were cytospun onto microscopy slides and stained with Rosenfeld solution. Morphological evaluations allowed for the frequencies of polymorphonuclear neutrophils and lymphocytes to be determined.

### 2.7. CFU Assay

Fragments of the lungs were disrupted via manual maceration in 1 ml of sterile PBS. 100 *μ*l of the samples was plated on BHI (brain–heart infusion agar; KASVI) supplemented with 5% fetal bovine serum (FBS) on Petri dishes and kept at 37°C for 20 days. Recovered colonies were counted, and results are expressed as mean log/g of organ.

### 2.8. Flow Cytometry

The supernatant of the disrupted lungs had its large particulate material removed with the use of a 70 *μ*m cell strainer. After osmotic lysis of red blood cells using solutions of different concentrations of NaCl (0.6% and 1.2%, respectively), cells obtained were suspended in PBS + 3% FBS and stained with monoclonal antibodies conjugated to fluorochromes allophycocyanin (APC), fluorescein isotiocyanate (FITC), or phycoerythrin (PE) following dilutions and protocol stated by the manufacturers. For this analysis, we used CD4 (clone RM4-4; FITC), CD8a (clone 53-6.7; PE), CD3 (clone 17-A2; APC), CD80 (clone 16-10A1; PE), CD11b (clone M1/70; FITC), GR1 (clone RB6-8C; APC), CD19 (clone MB19.1; APC), and NK1.1 (clone 1D3; PE), from BD Pharminger. We also used CD11c (clone N418; FITC) and MHC-II (clone AF6-120.1; APC) from Thermo Fisher. After washing, cells were transferred to FACS tubes and analyzed at FACSCanto (Becton and Dickinson). Strategy of gating was done according to the cell size (forward scatter) and internal complexity (side scatter) in order to exclude cellular debris and doublet readings. Data were analyzed using software FlowJo, and results were obtained as the percentage of double positive cells for specific labeling.

### 2.9. Organ Homogenates

The lungs, livers, and spleens of the mice were separately collected and disrupted in RIPA using a tissue homogenizer (Polytron). The supernatants were separated from the cellular debris by centrifugation at 12,000 rpm for 20 minutes, collected, and stored at −70°C. Protein concentration in homogenates was determined by commercial kits (bicinchoninic acid (BCA), Pierce, Rockford, IL, USA). The assay was performed according to the manufacturer's manual. Absorbance values were obtained at 562 nm (Varioskan Microplate Reader, Thermo Fisher Scientific).

### 2.10. Quantification of Cytokines

IL-4, IL-6, IL-10, TNF-*α*, and cytokine-induced neutrophil chemoattractant 1 (CINC-1) were determined in the BALF, the PeLF, and lungs, livers, and spleen homogenates by enzyme-linked immunoassay (ELISA) using commercial kits (R&D Systems Inc., Minneapolis, MN, USA). Assays were performed according to the manufacturer's manual. Data were converted to log prior to statistical analysis, and results were expressed in pg/ml.

### 2.11. Data Analysis

Data were processed and analyzed by analysis of variance (ANOVA) and the Tukey-Kramer post test or unpaired *t*-tests using GraphPad Prism 6.0 software (La Jolla, CA, USA). The two-tailed *p* values with 95% confidence intervals were acquired. Data are represented as mean ± standard error of mean (SEM). Values of *p* < 0.05 were considered significant.

## 3. Results

### 3.1. Diabetes Mellitus on *Paracoccidioides brasiliensis* Infection

**Figure 2:**
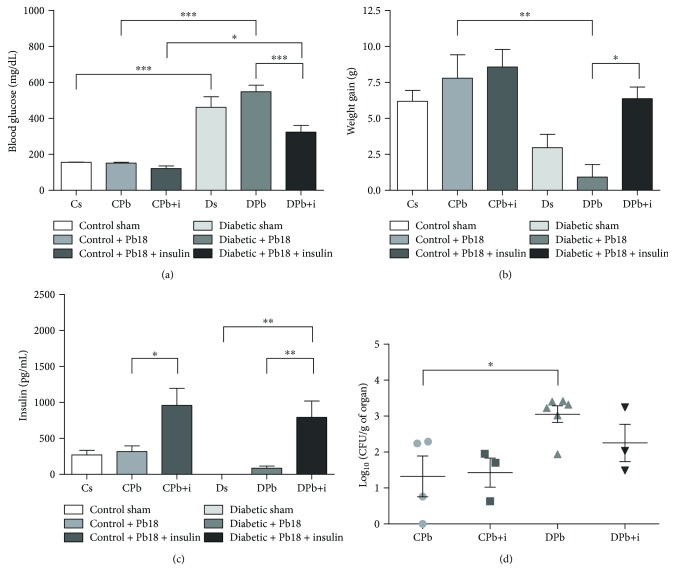
General characteristics of the animals. Mice were rendered diabetic via injection of alloxan (60 mg/kg i.v.) and infected with *P. brasiliensis* (1 × 10^6^ cells i.t.). Blood glucose levels and body weight gain in the diabetic and nondiabetic groups were determined 55 days after alloxan injection and again after the 45-day incubation period. Five animals were included in each group, and values are the means ± SEM. (a) Blood glucose levels after 55 days. (b) Body weight gain after 55 days. (c) Insulin levels in serum. (d) Colony-forming unit count. ^∗^*p* < 0.05; ^∗∗^*p* < 0.01; ^∗∗∗^*p* < 0.001.

To better evaluate the effects of insulin treatment during Pb18 infection in diabetic and nondiabetic mice, we first analyzed the characteristic parameters of type 1 diabetes mellitus throughout the experimental period. C57BL/6 mice were rendered diabetic by alloxan. Data in brackets represent mean ± SEM. After ten days, animals of the diabetic group presented higher blood glucose levels (570 ± 9.3 mg/dl vs. 192 ± 7.5 in the control group; *n* = 20; *p* < 0.001) and a significant reduction in body weight gain compared to the control group (0.87 ± 0.3 g vs. 2.94 ± 0.4 in the control group; *n* = 20; *p* < 0.001). Glycemia and body weight were measured again at the end of the experiment and at 45 days after Pb18 infection. We observed that the infection did not alter these parameters in both control and diabetic groups. Treatment with insulin (2 UI/600 mg/dl blood glucose) did not affect the glucose levels in the control infected group but reduced glucose levels in diabetic infected mice (Figure 2(a)), also increasing the weight gain (Figure 2(b)) in the diabetic infected group.

Insulin levels in the blood were determined to further characterize the model of experimental diabetes and subcutaneous insulin treatment. Animals in the diabetic group presented reduced insulin blood levels compared to the control group. Both the control and diabetic groups treated with insulin for 12 days presented higher levels of the hormone in plasma (Figure 2(c)).

CFU analysis was performed to determine the extent of fungal infection in the lungs of diabetic and nondiabetic mice. Diabetic mice from the infected group showed increased fungal load in lungs compared to nondiabetic infected mice. Treatment with insulin promoted no significant change on fungal load in nondiabetic animals (Figure 2(d)).

### 3.2. Cellularity in Blood, BALF, and PeLF

Total blood, BALF, and PeLF were analyzed for cellularity parameters that would help elucidate the chronic inflammation caused by Pb18 infection in diabetic and nondiabetic mice with or without insulin treatment.

**Figure 3:**
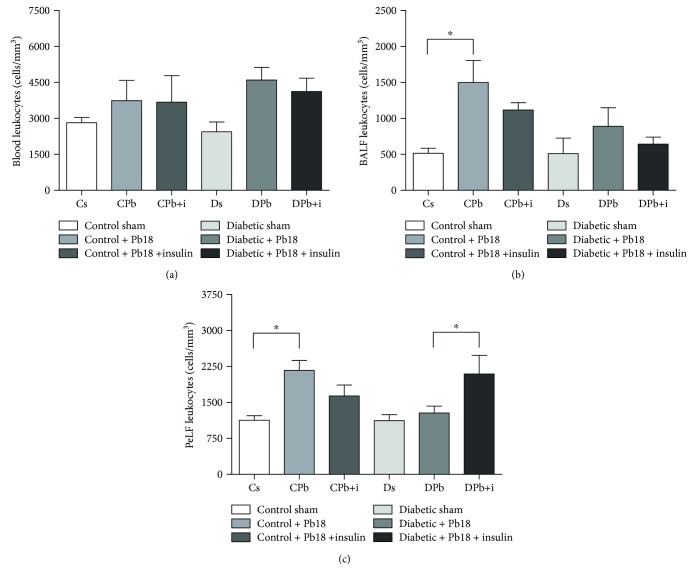
Blood/lavage fluid cellular analysis. Mice were rendered diabetic via injection of alloxan (60 mg/kg i.v.) and infected with *P. brasiliensis* (1 × 10^6^ cells i.t.). Samples were obtained 55 days after alloxan injection and 45 days after the incubation period. Five animals were included in each group. Values are the means ± SEM. (a) Leukocyte count in total blood. (b) Leukocyte count in BALF. (c) Leukocyte count in PeLF. ^∗^*p* < 0.05; ^∗∗^*p* < 0.01; ^∗∗∗^*p* < 0.001.

The infection with Pb18 did not cause significant alterations in leukocytes present in the total blood in samples obtained 45 days after infection in both diabetic and nondiabetic groups, nor did insulin treatment significantly change this parameter (Figure 3(a)). Analyzing BALF cellularity, we observed a 3-fold increase in the presence of inflammatory cells in the BALF of infected mice compared to controls (Figure 3(b)). An increase of migration of cells can also be observed in the PeLF of infected animals, characterizing an immunological response caused by the agent (Figure 3(c)). Diabetic infected mice did not present differences on cell migration in both PeLF and BALF compared to diabetic mice. Insulin treatment led to a higher leukocyte migration to the inflammatory exudate in PeLF of diabetic infected animals (Figure 3(c)), while this parameter did not change in BALF of diabetic infected mice receiving the treatment (Figure 3(b)).

### 3.3. Analysis of Leukocyte Populations in Lungs

**Figure 4:**
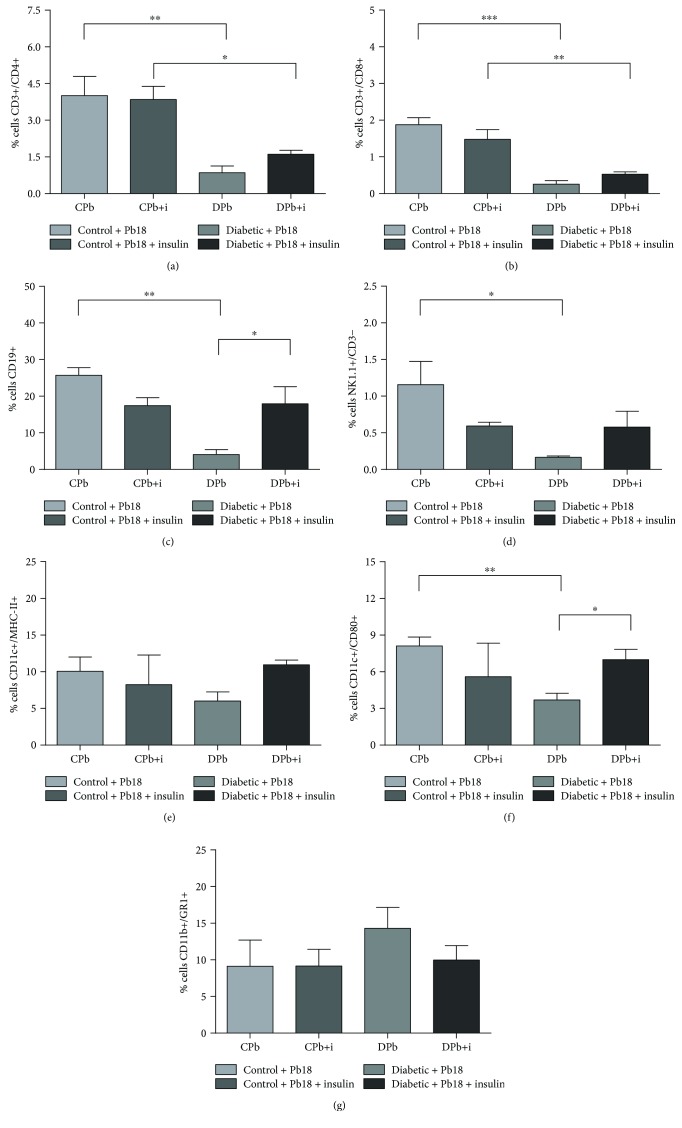
Leukocyte populations in lungs. Mice were rendered diabetic via injection of alloxan (60 mg/kg i.v.) and infected with *P. brasiliensis* (1 × 10^6^ cells i.t.). Lung samples were obtained 55 days after alloxan injection and 45 days after the incubation period. Five animals were used for each group. Values are the means ± SEM. ^∗^*p* < 0.05; ^∗∗^*p* < 0.01; ^∗∗∗^*p* < 0.001.

Lung cells were labeled by monoclonal antibodies, and readings were performed to determine differences in populations of cells migrating to the inflammatory focus in lungs. In samples collected after 45 days of incubation, diabetic mice presented a reduced influx of TCD4+ cells (CD3+/CD4+; Figure 4(a)), TCD8+ cells (CD3+/CD8+; Figure 4(b)), B lymphocytes (CD19+; Figure 4(c)), NK cells (NK1.1+/CD3−; Figure 4(d)), and dendritic cells (CD11c+/CD80+; Figure 4(f)) when compared to control infected groups.

Moreover, we see that 12 days of insulin treatment did not significantly alter the cell population in nondiabetic infected animals, while it restored the presence of B lymphocytes, dendritic cells, and NK cells in the lungs of diabetic animals (Figures 4(c), 4(d), and 4(f)).

### 3.4. Cytokine and Chemokine Analyses in Organ Homogenates

**Figure 5:**
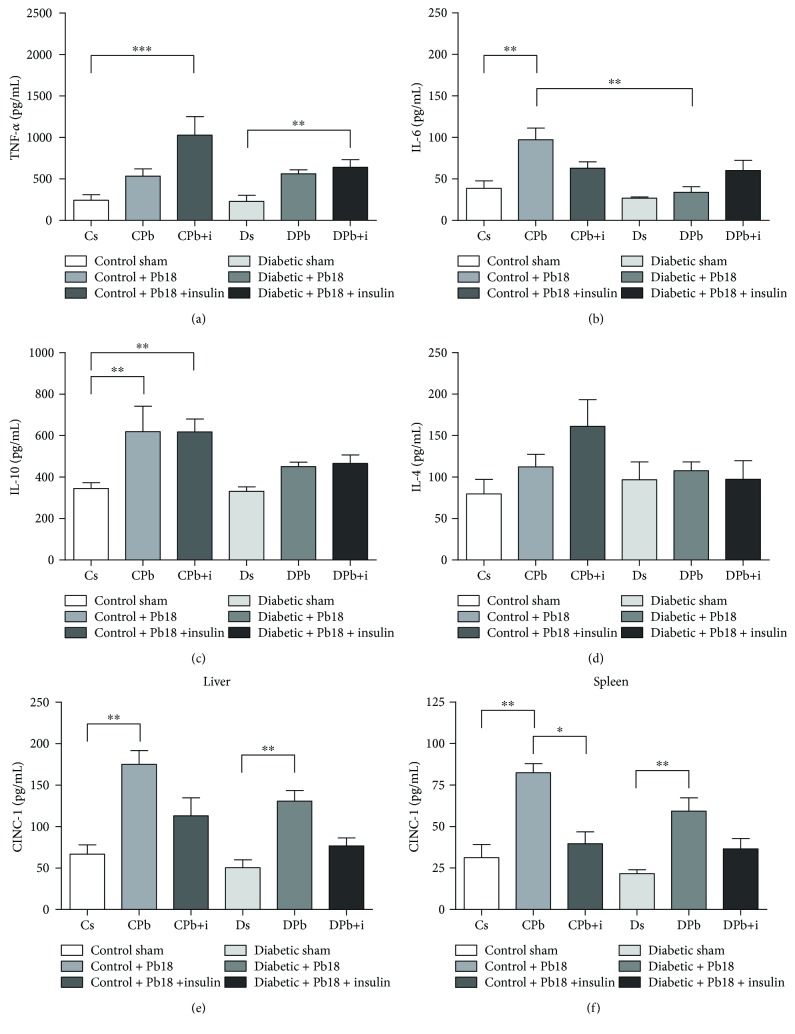
Cytokine and chemokine profiles in organ homogenates. Mice were rendered diabetic via injection of alloxan (60 mg/kg i.v.) and infected with *P. brasiliensis* (1 × 10^6^ cells i.t.). Tissue samples were obtained 55 days after alloxan injection and 45 days after the incubation period. Five animals were used for each group. Values are the means ± SEM. ^∗^*p* < 0.05; ^∗∗^*p* < 0.01; ^∗∗∗^*p* < 0.001. (a–d) Cytokine analysis in lung homogenates; (e) CINC-1 concentration in the liver; (f) CINC-1 concentration in the spleen.

Lungs, spleens, and livers were used to quantify cytokines to better understand the inflammatory condition from different sites of systemic infection. Pb18 infection induced higher concentrations of IL-10 and IL-6 in the lungs of control mice (Figures 5(b) and 5(c)); diabetic infected mice did not exhibit alterations in these levels. Insulin treatment did not induce increased cytokine production against the infection in diabetic mice, although mice from both the control group and the diabetic group infected by Pb18 showed increased production of TNF-*α* after insulin treatment (Figure 5(a)). This model of infection did not exhibit significantly altered cytokines in both spleen and liver homogenates (data not shown).

Chemokines in the spleens and livers were quantified to better understand the migration of inflammatory cells to different sites. Mice infected by Pb18 showed higher levels of CINC-1 in both liver and spleen homogenates (Figures 5(e) and 5(f), respectively). These levels were also increased in diabetic infected mice. However, animals from both diabetic and nondiabetic groups that received insulin treatment exhibited reduced secretion of CINC-1 in both sites (Figures 5(e) and 5(f), respectively).

## 4. Discussion

The data presented here suggest that insulin modulates inflammatory parameters in the systemic mycosis Pb18 model in diabetic and nondiabetic mice. DM represents a group of diseases with a continually increasing global incidence, clinical relevance, and economic impact [16]. It has been suggested that DM1 causes negative effects on the quality of the patient's immune response [26], as evidenced by reduced interaction between leukocytes and endothelium and reduced migration of inflammatory cells rendering diabetic patients more susceptible to infections that affect mucosa, respiratory, and urinary traits [27]. In some studies, a lack of insulin resulting from DM1 evolution was suggested to be responsible for impaired immunological responses rather than the excess blood glucose [22].

The experimental DM model developed using alloxan is based on the cytotoxic effects of alloxan in *β*-pancreatic cells, where intercellular lesions caused by reactive oxygen species destroy cells promoting a diabetic state [28]. The data shown in this study corroborate this experimental model: i.v. alloxan administration to male mice produced all the symptoms of a diabetic state within 10 days—elevated concentrations of blood glucose, reduced body weight gain compared to nondiabetic animals, and reduced concentrations of insulin in plasma, among other characteristic effects of the disease, such as elevated consumption of water and higher urine volume. The induction of type 1 diabetes by injection of alloxan, without any kind of stimulus, does not promote significant alteration on inflammatory parameters when compared to the control saline animals. For this study, we selected the insulin treatment reported by Spiller et al. [24] and calculated the insulin dose without reducing the diabetic animals' glucose levels below 300 mg/dl.

The inflammatory stimulus chosen for this study was chronic inflammation and systemic mycosis caused by Pb18. As occurring in most fungal infections, the proper function of macrophages/monocytes is extremely important for innate mechanisms to control this infection. Both TCD4+ and TCD8+ cells are reported to play a major role in the control of Pb infection through the formation of granulomas that will contain the replication of the pathological agent [4, 14]. This shows that the type of inflammatory response is equally important, as the progression of the disease is characterized by reduction of Th1 cytokines (IL-12, IFN-*γ*) resulting in reduced activation of TCD4+ and TCD8+ cells, together with a higher production of IL-4 and IL-10 and an augmented presence of B lymphocytes [29].

In samples obtained 45 days after infection and 12 days after insulin treatment, we observed that the diabetic mice exhibited maintained diabetic parameters through incubation of the fungus. The presence of the fungus did not promote significant changes in these parameters in the diabetic and nondiabetic animals, although the data suggest that the body weight gain was even more evident in the diabetic animals infected by Pb18. The insulin treatment produced no effect in the nondiabetic mice, whereas diabetic infected mice treated with insulin gained significantly more weight compared to the other diabetic groups. Moreover, we observe that diabetic mice were more susceptible to the infection on a long term, as they show higher fungal load suggesting reduced capability of controlling the proliferation of fungi when compared to nondiabetic mice. The CFU count was slightly reduced in diabetic animals that received the treatment, suggesting that insulin permitted a more effective immunological response against the pathological agent, although the insulin dose was not enough to normalize the glucose levels in the diabetic mice.

Cell migration plays a crucial role in immunological responses and the expression of adhesion molecules, allowing leukocytes to leave blood vessels to access the site of inflammation. In a Pb infection, the migration and activation of macrophages and TCD4 and TCD8 cells are described to have crucial importance on stopping the proliferation of the etiological agent, thus limiting the infection. A higher number of leukocytes in the BALF of animals infected by the fungus were observed, suggesting the in situ inflammatory process. Suggestion of systemic infection can be observed by the increased number of leukocytes found in the PeLF of infected animals. However, the diabetic infected mice presented fewer leukocytes in both the BALF and PeLF; insulin treatment restored leukocyte migration to the PeLF but not to the BALF. This could be explained by the reduced expression of adhesion molecules, fungicidal activity, and production of inflammatory cytokines by macrophages [24]. Moreover, several features may influence migration of leukocytes to the inflammation focus during a fungal infection. Taylor et al. evidenced in 2006 the importance of a collaborative response between dectin-1 and Toll-like receptors to induce a response able to recruit lymphocytes resulting in the containment and killing of the fungi. Likewise, dectin-1-knockout mice presented reduced leukocyte migration to the peritoneal cavity during infection of *Candida albicans*, resulting also in higher dissemination of the fungi and a higher mortality rate [30]. Souto and associates also showed the proinflammatory cytokine IFN-*γ* to have an essential involvement with the modulation of leukocyte recruitment during a lung infection of Pb: compared to wild-type mice, mice deficient of IFN-*γ* presented reduced migration of macrophages and lymphocytes while causing accumulation of neutrophils on the lungs. Albeit it does not alter the total leukocyte concentration, the change of the inflammatory profile rendered the mice more susceptible to the infection [31].

In addition, our data show that diabetic infected mice presented reduced migration of TCD4 and TCD8 cells, NK cells, dendritic cells, and B lymphocytes, which would allow a less effective immunological response and allow the agent to proliferate, as evidenced by a higher fungal load obtained in CFU analysis. B lymphocytes (CD19+) are described as necessary for host resistance in murine models of Pb infection. Tristão and associates showed in 2013 that the deficiency of B lymphocytes in Pb-infected mice resulted in a lower survival rate and higher fungal load in lungs [32]. This and other studies report the involvement of B lymphocytes on the control of the pathological agent's growth and on the organization of the granulomas in granulomatous diseases [33, 34]. In our work, the treatment with insulin on diabetic infected mice resulted in not only augmentation of the B lymphocyte population but also an enhanced antigen-presenting ability, as evidenced by higher concentrations of the costimulatory molecule CD80 in antigen-presenting cells. This suggests insulin to be modulating the microenvironment to a more protective role to the organism, as the role of those cells tends to prevent the spreading of the fungi rather than prioritizing its destruction.

Siqueira et al. showed in 2009 the relation between higher levels of IL-6 and successful infection by Pb, where IL-6 modulates other cytokines to ensure the growth of the pathogenic agent [35]. In addition, Tristão et al. described IL-6 and IL-23 as essential cytokines to produce the inflammatory profile needed to induce the formation of granulomas, limiting the spreading of the fungi [36]. Here, the infection induced by Pb18 caused augmented production of IL-10 and IL-6 by the lung tissue of infected animals from the nondiabetic groups. Diabetic infected mice did not show increased levels of these cytokines, indicating a state of higher susceptibility to the Pb infection and its outcomes. Insulin treatment, while altering other parameters that would help in a more efficient containment of the infection, did not promote any changes on the studied cytokines. Interestingly, insulin treatment resulted in enhanced production of TNF-*α* by infected nondiabetic mice, followed by a reduction of IL-6 levels. The inversed relation of TNF-*α* and IL-6 levels has also been described as a defense mechanism of the fungus: IL-6 can inhibit the production of TNF-*α*, which is directly related to macrophage function in PCM models and acts on granuloma formations and the modulation of fungicidal activity [4, 6, 35].

The higher concentrations of the chemokine CINC-1 were observed in both spleen and liver homogenates of both diabetic and nondiabetic infected mice. CINC-1 is a potent chemotactic agent that has been described as related to neutrophil migration to inflammatory sites. Both diabetic and nondiabetic mice treated with insulin presented reduced levels of CINC-1 in liver and spleen homogenates, suggesting that the enhanced presence of leukocytes in the PeLF was not due to migration.

The data presented here suggest that diabetic mice present impaired immunological response to 45 days of a Pb18 infection and that insulin modulates Pb18-induced inflammation by restoring the populations of dendritic cells and B lymphocytes in lungs, permitting the host to a more effective control of the infection.

## Data Availability

The values behind the means, standard deviations, and other measures reported in the data supporting the findings of this study can be obtained from the corresponding author upon reasonable request (Joilson O. Martins, martinsj@usp.br).
